# Remote reprogramming of hepatic circadian transcriptome by breast cancer

**DOI:** 10.18632/oncotarget.16699

**Published:** 2017-04-06

**Authors:** Hiroaki Hojo, Sora Enya, Miki Arai, Yutaka Suzuki, Takashi Nojiri, Kenji Kangawa, Shinsuke Koyama, Shinpei Kawaoka

**Affiliations:** ^1^ Advanced Telecommunications Research Institute International (ATR), The Thomas N. Sato BioMEC-X Laboratories, Kyoto, Japan; ^2^ ERATO Sato Live Bio-Forecasting Project, Japan Science and Technology Agency (JST), Kyoto, Japan; ^3^ The University of Tokyo, Graduate School of Frontier Science, Kashiwa, Japan; ^4^ Department of Biochemistry, National Cerebral and Cardiovascular Center Research Institute, Suita, Japan; ^5^ Department of General Thoracic Surgery, Osaka University Graduate School of Medicine, Suita, Japan; ^6^ Department of Statistical Modeling, Institute of Statistical Mathematics, Tokyo, Japan

**Keywords:** breast cancer, RNA-Seq, hepatic circadian transcriptome, hepatic oxidative stress, hepatic polyploidization

## Abstract

Cancers adversely affect organismal physiology. To date, the genes within a patient responsible for systemically spreading cancer-induced physiological disruption remain elusive. To identify host genes responsible for transmitting disruptive, cancer-driven signals, we thoroughly analyzed the transcriptome of a suite of host organs from mice bearing 4T1 breast cancer, and discovered complexly rewired patterns of circadian gene expression in the liver. Our data revealed that 7 core clock transcription factors, represented by *Rev-erba* and *Rorg*, exhibited abnormal daily expression rhythm in the liver of 4T1-bearing mice. Accordingly, expression patterns of specific set of downstream circadian genes were compromised. *Osgin1*, a marker for oxidative stress, was an example. Specific downstream genes, including *E2f8*, a transcriptional repressor that controls cellular polyploidy, displayed a striking pattern of disruption, “day-night reversal.” Meanwhile, we found that the liver of 4T1-bearing mice suffered from increased oxidative stress. The tetraploid hepatocytes population was concomitantly increased in 4T1-bearing mice, which has not been previously appreciated as a cancer-induced phenotype. In summary, the current study provides a comprehensive characterization of the 4T1-affected hepatic circadian transcriptome that possibly underlies cancer-induced physiological alteration in the liver.

## INTRODUCTION

Cancer is a disease that affects host physiology at multiple levels, worsening quality of life and ultimately in certain cases causes organismal death. Such physiological disruption includes multi-organ chronic inflammation, insulin resistance, metabolic disorder, cachexia, and so on [1, 2]. However, mechanisms underlying these cancer-mediated physiological alterations and their physiological significance are still poorly understood. Moreover, it remains enigmatic how these physiological disturbances are related to each other. Understanding the nature of cancer-induced physiological disruption on a host/patient, especially at an earlier phase of cancer progression *in vivo*, may help early detection and prevention of cancers. Most importantly, mitigating cancer-induced adverse effects on host physiology, if possible, may provide patients a way to live with cancer while maintaining a relatively high quality of life.

Animal physiology is under the tight control of the circadian clock. Circadian clocks are cell-autonomous oscillators that drive rhythmic gene expression, and are responsible for daily rhythms of animal physiology, behavior and metabolism. In mammals, the hypothalamic suprachiasmatic nucleus (SCN) functions as the master pacemaker to regulate the whole-organismal rhythm [3]. Peripheral organs harbor their own autonomous circadian clocks but are synchronized by the SCN via neural and endocrine pathways [4]. Yet, these two distinct circadian machineries employ similar molecular devices to generate daily rhythms of gene expression [5]. Transcription factors Clock and Bmal1 hetero-dimerize to activate expression of Period (Per1/2/3) and Cryptochrome (Cry1/2) via the E/E´ box sequence. Per-Cry protein complexes in turn inhibit the transcription-activating role of Clock/Bmal1, decreasing *Per* and *Cry* expression, thereby forming the negative feedback loop. Additional components such as Rev-erbα and Rorγ also contribute to establishing proper circadian rhythms and are thought to have a role in establishing the night-time transcriptome [5]. These timely expressed transcriptional activators and repressors, and their target *cis*-elements E/E'-box, D-box, and RRE-box sequences define the transcriptomes of “morning”, “day-time”, and “night-time”, respectively [6]. In the liver as an example, more than 1,000 genes oscillate [7, 8]. Although the SCN-based central circadian clock acts upstream of peripheral clocks, peripheral oscillators are at the same time independently modulated locally [4]. These mechanisms sophisticatedly achieve a context-dependent tuning of circadian rhythms at local and systemic levels, enabling organisms to adapt their physiological state to the external environment over the course of a day.

Circadian rhythms can be modulated or disrupted by numerous endogenous and exogenous factors. For example, temporal feeding restriction alters circadian gene expression in peripheral tissues such as the liver, while leaving those in the SCN unaffected [9, 10]. Similarly, a high-fat diet (HFD) interferes with circadian transcriptome and metabolome in the liver [11]. These findings suggest that feeding *per se* and food composition directly influences peripheral circadian rhythms. Furthermore, signaling molecules impact circadian rhythm: activation of TGFβ signaling results in phase-shifts of a set of oscillating genes in the kidney and adrenal gland [12]. The TGFβ-dependent phase-shift genetically requires a transcription factor Dec1 that suppresses Clock/Bmal1-regulated *cis*-elements [12]. Importantly, in a normal physiological condition, organisms are able to “tune” their circadian rhythms predominantly by light, which controls the SCN-centered clock through the retina and the retinohypothalamic tract [13]. Chronically disrupted circadian rhythm potentially causes diseases including metabolic disorder and cancer [14–16]. However, the causative role for disrupted circadian rhythms in diseases is still unclear, especially due to technical difficulties to experimentally cure disturbed circadian rhythms in a specific manner.

Cancer tissues secrete a number of cytokines and hormones, possibly affecting systemic circadian rhythm. Indeed, using a genetically engineered mouse model of lung adenocarcinoma driven by a mutant version of Kras (Kras^G12D^) and loss of a tumor suppressor *p53*, Masri et al. recently showed that the lung adenocarcinoma rewires hepatic circadian rhythm both at the transcriptome level and metabolome level, curiously without affecting the core clock transcription factors for instance *Clock* [17]. Although the study has revealed the intriguing connection between the lung adenocarcinoma and hepatic circadian rhythm, several major questions are still unanswered. Is the rewired hepatic circadian rhythm physiologically important for other cancer-induced phenotypes e.g chronic inflammation? Is the cancer-induced rewiring of hepatic circadian rhythm at the genome-wide level a general phenomenon? Is there cancer-type specific circadian alteration? Answering these important questions will extend our understanding on circadian clocks and the rhythms they generate as a target of cancer-induced physiological disruption.

Here we show that a mouse model of triple negative breast cancer, 4T1, remotely interferes with hepatic circadian gene expression patterns. Through extensively investigating the effects of 4T1 breast cancer on hepatic gene expression, we revealed that 4T1 disrupted daily expression patterns of 7 core clock transcription factors and a number of downstream circadian genes. Some in those exhibited liver-specific alteration. We identified a particular set of genes that completely lost their original daily oscillation. These disruption patterns were distinct from those observed in mice harboring the lung adenocarcinoma [17]. Our RNA-seq data indicated that the liver of 4T1-bearing mice suffered from oxidative stress and anomalous hepatic polyploidy, the notion validated by histological and flow cytometric analyses. Our data demonstrate a general role of solid cancer in reprogramming hepatic circadian clocks, and at the same time, shed light on cancer type-specific disruption patterns of circadian transcriptomes.

## RESULTS

### Transcriptome analyses on 4T1-affected gene expression in multiple distant organs

**Figure 1:**
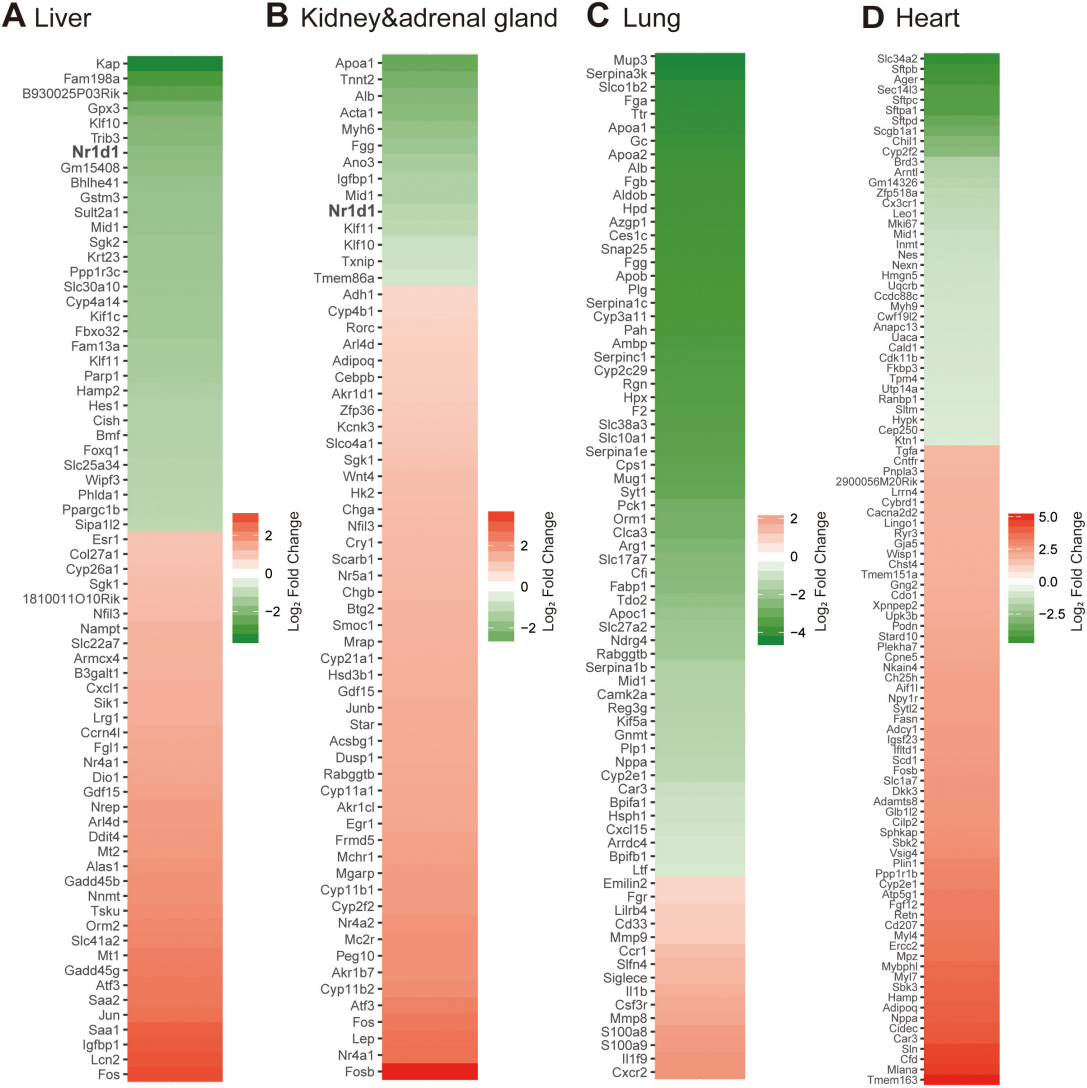
Transcriptome analyses on 4T1-affected gene expression in multiple distant organs **(A)-(D)** Heatmaps showing genes significantly affected by 4T1 transplantation in the liver **(A)**, kidney and adrenal gland **(B)**, lung **(C)** and heart **(D)**. Data from D3 and D7 post transplantation were analyzed as biological replicates. Essentially all of significantly altered genes are visualized except for up-regulated genes in the heart: 72 of 275 up-regulated genes are indicated in the heatmap. The full-lists of significantly affected genes accompanied with expression data are provided in Supplementary Tables 1-8.

4T1 is a transplantable mammary carcinoma cell line that can grow *in vivo* as primary cancer. 4T1 harbors characters (e.g. metastasis pattern) similar to human mammary carcinoma and thus has been used for breast cancer researches [18]. Using this model, we attempted to identify host genes that respond to 4T1 transplantation at earlier time points (i.e. before metastasis). For this purpose, the liver and lung, the two major targets of 4T1 metastasis [19–21], kidney (including the adrenal gland), and heart were chosen for RNA-seq analyses. 4T1-bearing or sham-operated mice were sacrificed at 3 and 7 days post-transplantation (dpt), when the extent of 4T1-induced inflammation measured by qRT-PCR against an inflammation marker *S100a8* was relatively mild (Supplementary Figure 1A). Importantly, visible metastases were not detected at these time points, an observation supported by histological analyses (Nojiri and Arai et al., submitted). The scores obtained from 3 dpt and 7 dpt were regarded as replicates to find significantly altered genes upon 4T1 transplantation (Figures 1A-1D and Supplementary Tables 1-8).

Each organ demonstrated unique gene expression changes upon 4T1 transplantation. Marked elevation for inflammatory signatures represented by *S100a8* in the liver and lung but not in the heart and kidney confirmed that the former two organs are preferred targets of cancer-induced inflammation [20]. Particularly, reduced expression of hepatic *Nr1d1*, also known as *Rev-erbα*, got our attention. Rev-erbα is a critical core clock component that maintains circadian rhythm as a morning-to-daytime-expressed repressor to define night-time transcriptome, and its expression is ubiquitous [22]. The decrease of hepatic *Rev-erbα* expression upon 4T1 transplantation was confirmed by qRT-PCR experiments (Supplementary Figure 1B). The altered expression of hepatic *Rev-erbα* was detected at the earliest time point of our experimental setting (Figure 1A and Supplementary Figure 1B), before massive inflammation and liver metastasis took place. We further found that the above-described gene expression changes were recapitulated in 1st-recipient mice directly injected with 4T1 cancer cell suspension, and thus we analyzed 1st-recipient mice for the following experiments. Together, these results led us to hypothesize that 4T1 cancer disrupts hepatic circadian rhythm.

### 4T1 cancer distantly disrupts the hepatic core clocks

**Figure 2:**
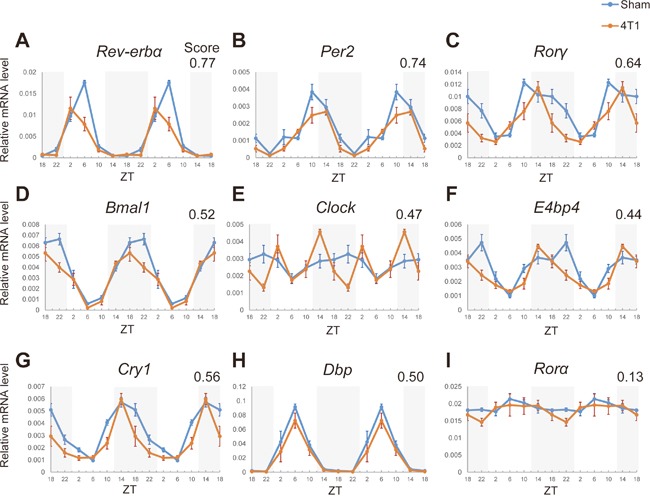
4T1 cancer disrupts the hepatic core clocks **(A)-(I)** Day time expression of the core clock genes, *Rev-erbα*, *Clock*, *Bmal1*, *Per2*, *Cry1*, *Rorα*, *Rorγ*, *Dbp*, *and E4bp4* in the livers of sham-operated (Sham) and 4T1-bearing (4T1) mice, determined by qRT-PCR. Mice were sacrificed on 7 dpt at the indicated time points. The daily expression patterns were double-plotted. Data are presented as the mean ± SE (n = 8 for ZT22 of the sham samples, n = 9 for ZT18, 2, 6, 10, 14 of the sham samples, and n = 6 for the 4T1-bearing samples). Average value of absolute log_2_ fold changes is indicated in the upper-right corner.

The reduction in *Rev-erbα* expression was observed approximately between at ZT2-ZT6 (ZT: zeitgeber time). This promoted us to explore whether *Rev-erbα* and other core clock components were affected at different ZTs. To this end, mice were injected with 4T1 cancer cells, sacrificed at 7 dpt at day-time (ZT2, ZT6, and ZT10) and night-time (ZT14, ZT18, and ZT22). qRT-PCR analyses were performed against 9 core clock transcription factors (Figure 2). To evaluate the effects of 4T1 cells on these core factors in the liver, we ranked the surveyed genes based on averages of absolute log_2_ fold changes at 6 ZTs: more than 1.5-fold changes on average (i.e. absolute log_2_ fold change > 0.585) were primarily considered to be important. When a gene showed less than 1.5-fold change on average, more than 1.5-fold changes with *p* < 0.05 on a specific time point (especially at a peak time) were taken into account. Genes that met both were expected to be a promising primary target of 4T1 cancer cells.

The daily expression patterns of the surveyed genes are shown in Figure 2 with the averages of absolute log_2_ fold changes. Striking alteration was observed for *Rev-erbα* and *Rorγ* (Figure 2). Expression of *Rev-erbα* was more than 2-fold decreased at the peak time (ZT6), and more than 1.5-fold difference on average was detected (Figure 2A). Since the most prominent change occurred at the peak time, this difference appeared to be meaningful. *Rorγ* exhibited significant reduction at the peak time (ZT10) and more apparent abnormality was observed at ZT22: rapid reduction after reaching at the peak (Figure 2C). These abnormalities can be written as a phase-delay in an activation phase and a phase-advance in a repression phase. These two genes were ranked within the top3-affected genes according to the averages of absolute log_2_ fold change score (0.77 for *Rev-erbα* and 0.64 for *Rorγ*). *Per2* was also ranked as the top3, but the difference at the peak time was relatively mild (1.54-fold) (Figure 2B). Despite not satisfying our criteria, a rewired expression pattern of *Clock* in 4T1-bearing mice was unique: occurrence of de novo abnormal peak at ZT14 and reduction at ZT22 (more than 1.5-fold change with *p* < 0.05 both at ZT14 and ZT22) (Figure 2E).

Although not top-ranked, the following differences were also observed. *Bmal1*, and *E4bp4* displayed significant decrease in their expression at ZT22 (Figures 2D and 2F). *E4bp4* exhibited a clear phase-advance (Figure 2F). While *Cry1* had no difference at the peak time (ZT14), its expression at ZT18-ZT22 in 4T1-bearing mice was more than 1.5-fold lower (*p* < 0.05) than the controls (Figure 2G). Altogether, we revealed that 4T1 breast cancer cells affected the expression patterns of 7 core clock genes in the liver. On the other hand, the kidney core clocks differently responded to 4T1 transplantation (Supplementary Figure 2): no surveyed core clocks met our criterion, and only *Rev-erbα* showed clear abnormality at the peak time (1.86-fold reduction, *p* = 0.003) (Supplementary Figure 2B). Expression of *Clock*, which was uniquely disrupted in the liver, was almost unaffected in the kidney of 4T1-bearing mice (Figure 2E and Supplementary Figure 2I). These results indicated that there is a liver specific program that reacts to 4T1 transplantation.

### Hepatic circadian gene expression downstream of the core clocks is compromised by 4T1 transplantation

Next we examined downstream alteration of oscillating genes in the liver by comprehensively analyzing hepatic transcriptomes at different times of day. RNA-seq experiments were performed against the livers of sham-operated or 4T1-transplanted mice (n = 2 each) at 6 ZT points (in total 24 samples) (Supplementary Tables 9-10). The obtained RNA-seq data were subjected to JTK cycle analysis [23] to identify rhythmic genes (Supplementary Table 11). Moreover, to make the following analysis more solid, we compared the rhythmic genes from our dataset with the previously published dataset [7], and listed 332 most reliably cycling genes in the liver (see materials and methods for details, and Supplementary Table 12).

**Figure 3:**
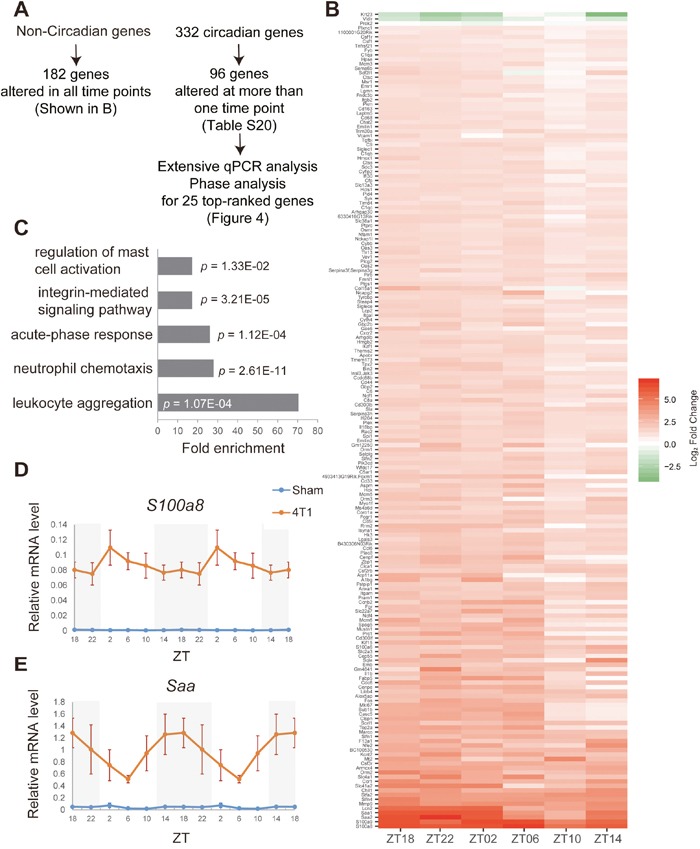
Transcriptome analyses on daily expression of hepatic genes in 4T1-bearing mice **(A)** The summary and analytic pipelines of 4T1-affected non-circadian genes and circadian genes. **(B)** The heatmap representation of highly significantly altered non-circadian genes. **(C)** Gene ontology analysis of the genes shown in **(B)**. **(D)-(E)** Daily expression patterns of *S100a8* and *Saa* in the liver of sham-operated (Sham) and 4T1-bearing (4T1) mice, as determined by qRT-PCR. Mice were sacrificed on 7 dpt at indicated time points. The daily expression patterns were double-plotted. Data are presented as the mean ± SE (n = 8 for ZT22 of the sham samples, n = 9 for ZT18, 2, 6, 10, 14 of the sham samples, and n = 6 for the 4T1 samples).

We also defined constitutively expressed non-circadian genes in the liver. These genes were considered to be circadian genes neither in our dataset (Supplementary Table 11) nor in [7]. Among them, 182 genes were significantly altered by 4T1 transplantation at all time points tested (Figures 3A-3B and Supplementary Table 13). This group of genes included inflammatory response genes such as *S100a8*, *Saa*, *Il1α*, and so on, supported by gene ontology (GO) analysis (Figure 3C and Supplementary Table 13). qRT-PCR experiments confirmed that *S100a8* and *Saa* were up-regulated throughout the day upon 4T1 transplantation (Figures 3D-3E). This demonstrated that, consistent with previous studies, breast cancer cells as well as other types of cancer induced distant inflammation in the liver [17, 20, 21].

We then investigated how 4T1 transplantation affected hepatic circadian gene expression (Supplementary Tables 14-19). Among the 332 oscillating genes (Supplementary Table 12), 96 genes were significantly altered at least at one ZT point (Supplementary Table 20). Importantly, *Rev-erbα, Per2, Clock, and Rorγ* were included in this group. This was consistent with our previous qRT-PCR data (Figure 2), and corroborated our findings.

**Figure 4:**
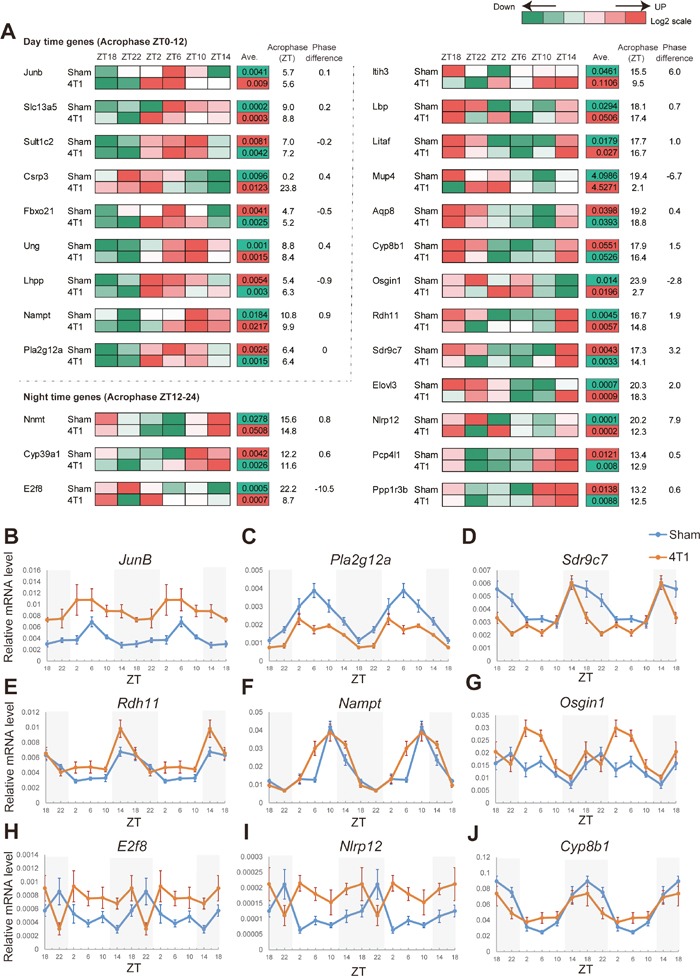
4T1 cancer remotely rewires expression of oscillating genes downstream of the hepatic core clocks **(A)** The heatmap representation of the rewired circadian gene expression in the liver of 4T1-bearing mice. The data were obtained by qRT-PCR analysis (n=6-9 for each time point). The averages of expression levels during the day are also shown. The acrophases (ZT) for each gene, and phase differences between sham-operated group and 4T1-transplanted group are indicated. The data for 25 genes (9 day-time genes and 16 night-time genes) are shown. **(B)-(J)** Daily expression patterns of *JunB*, *Pla2g12a*, *Sdr9c7*, *Rdh11*, *Nampt, Osgin1*, *E2f8*, *Nlrp12*, and *Cyp8b1* in the liver of sham-operated (Sham) and 4T1-bearing (4T1) mice, as determined by qRT-PCR. The daily expression patterns are double-plotted. Data are presented as the mean ± SE (n = 8 for ZT22 of the sham samples of *Nampt*, *Osgin1*, *E2f8*, *Nlrp12*, and *Cyp8b1*, n = 9 for ZT18, 2, 6, 10, 14 of the sham samples of *Nampt, Osgin1*, *E2f8*, *Nlrp12*, and *Cyp8b1*, and n = 6 for other samples).

To investigate the characteristics of hepatic circadian alteration in 4T1-bearing mice in more details, qRT-PCR experiments were performed against a set of oscillating genes (n = 6-9 for each time point). From 96 4T1-affected circadian genes (Figure 3A and Supplementary Table 20), we analyzed (i) all of 18 downstream genes whose expressions were significantly altered at least at 3 ZT points in 4T1-bearing mice, and (ii) 7 out of 17 genes whose expression was significantly altered at 2 ZT points (Figure 4A). In addition, an acrophase of each gene was determined by fitting a sinusoidal function to qRT-PCR data [24]. Accordingly, we classified 25 genes into 9 day-time genes and 16 night-time genes (Figure 4A). Averages of expression levels of 6 ZT points were also plotted to compare overall mRNA abundance (Figure 4A).

Figure 4 clearly showed that a phase shift phenotype was somewhat specific to a set of genes (Figure 4). *JunB* was up-regulated during the day in 4T1-bearing mice, while the acrophase was not affected (Figure 4B). Similarly, expression of *Pla2g12a*, *Sult1c*, and *Pcp4l1* was reduced throughout the day while the peak times were unaffected (Figure 4C and Supplementary Figure 3A-3B). In contrast, our data highlighted *Sdr9c7*, *Rdh11*, *Nampt*, *Elovl3*, and *Osign1* as “phase-affected genes.” The expression pattern of *Sdr9c7* was phase-advanced by approximately 3 h (Figure 4D). *Rdh11, Nampt, and Elovl3* also displayed phase advances (Figures 4E-4F and Supplementary Figure 3C), and *Osgin1* showed a phase-delay (Figure 4G). Most importantly, we discovered that 3 night-time genes entirely lacked their original oscillations: *E2f8*, *Nlrp12*, and *Cyp8b1* (Figures 4H-4J). *E2f8*, *Nlrp12* and *Cyp8b1* were always abnormally expressed throughout the day, and *E2f8* and *Nlrp12* exhibited marked “day-night reversals.” In contrast to these alterations, many other circadian genes appeared to be unaffected by 4T1 transplantation, as represented by *Dec1* expression (Supplementary Figure 3D).

Collectively, our analyses demonstrated that unique set of genes in the liver exhibited altered expression patterns or completely lost their circadian rhythms upon 4T1 transplantation. The list of 4T1-affected circadian genes were distinct from those described in [17], suggesting that different solid cancer rewires distinct module of hepatic circadian clocks.

### The liver of 4T1-bearing mice suffered from inflammation, oxidative stress, and altered polyploidy

**Figure 5:**
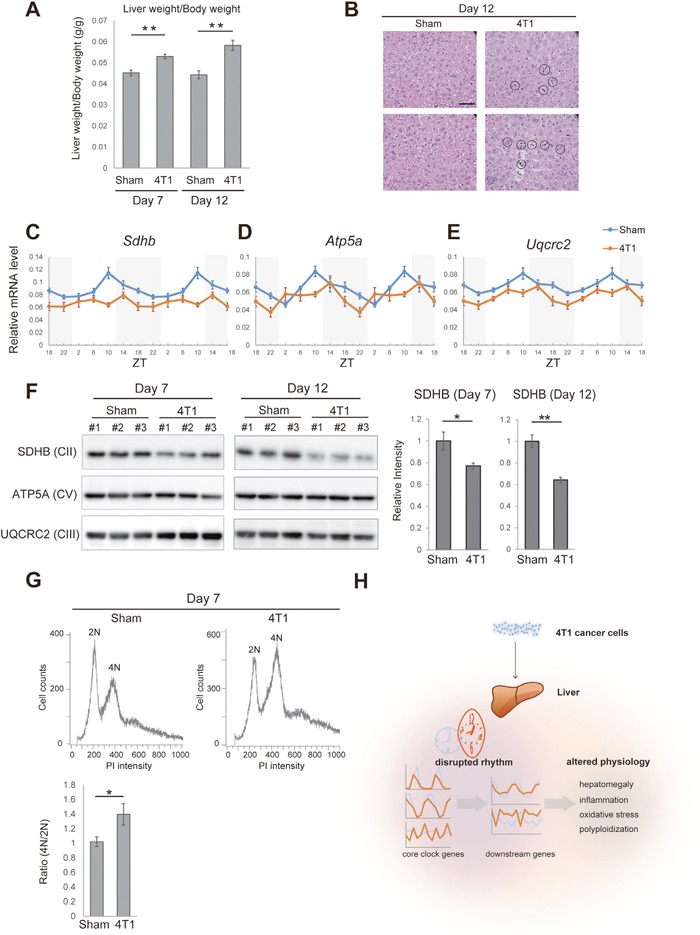
The liver of 4T1-bearing mice exhibits increased oxidative stress and altered polyploidy **(A)** The liver weight of sham-operated (Sham) and 4T1-bearing (4T1) mice, which is normalized by the body weight, on the indicated day points. Data are presented as the mean ± SE (n = 4), and asterisks indicate statistical significance as determined by Student's *t*-test; **p < 0.005. **(B)** Histological analysis of the liver of sham-operated (Sham) and 4T1-bearing (4T1) mice on 12 dpt. The scale bar represents 50 μm. Circles indicate massive accumulation of leukocytes. **(C)-(E)** Daily expression of *Sdhb*, *Atp5a1*, and *Uqcrc2* in the liver of sham-operated (Sham) and 4T1-bearing (4T1) mice, as determined by qRT-PCR on 7 dpt at the indicated time points. The daily expression patterns are double-plotted. Data are presented as the mean ± SE (n = 6). **(F)** Western blotting for subunit proteins of mitochondrial respiratory complexes (SDHB, ATP5A1 and UQCRC2) in the liver of sham-operated (Sham) and 4T1-bearing (4T1) mice on 7 (n = 3 each) and 12 dpt (n = 3 each). Quantification for SDHB intensity is also shown. Data are presented as the mean ± SE (n = 3), and asterisks indicate statistical significance as determined by Student's *t*-test; **p < 0.005 and *p < 0.05. **(G)** Representative flow cytometry data showing the DNA content of hepatocytes on 7 dpt. Tetraploid cells (4N) and diploid cells (2N) are indicated. Quantification for the 4N/2N ratio is also shown. Data are presented as the mean ± SE (n = 3), and asterisks indicate statistical significance as determined by Student's *t*-test; *p < 0.05. **(H)** Schematic overview depicting the effects of 4T1 cancer on hepatic circadian rhythm and liver physiology.

Finally, we asked whether these gene expression changes were related to detectable alteration in the liver physiology. The liver of 4T1-bearing mice was enlarged, a phenomenon known as hepatomegaly (Figure 5A). In accordance with Figure 3, histological analyses demonstrated that a number of leukocytes had infiltrated into the liver of 4T1-bearing mice (Figure 5B). Concurrently, we detected increase for *Osgin1* (also known as *OKL38*), a marker for oxidative stress (Figure 4G) [25]. To further assess whether the liver of 4T1-bearing mice was under increased oxidative stress, we conducted qRT-PCR analyses against a set of subunit factors of mitochondrial respiratory complexes. As shown in Figures 5C-5E, *Sdhb* (the gene coding a subunit of complex II) and *Uqcrc2* (a subunit of complex III) were slightly down-regulated in the liver of 4T1-bearing mice throughout the day. *Atp5a1* (a subunit of complex V) exhibited a disturbed daily oscillation pattern upon 4T1 transplantation (Figure 5E). These results were validated at the protein level by western blotting using anti-OXPHOS antibodies. As shown in Figure 5F, SDHB was significantly reduced in the liver of 4T1-bearing mice whereas no detectable reduction for UQCRC2 and ATP5A was observed on 7 and 12 dpt (Figure 5F). Although why SDHB was particularly sensitive to 4T1 transplantation remains unclear, we concluded that the liver of 4T1-bearing mice was inflamed and suffered from oxidative stress.

It has been known that increased oxidative stress in the liver promotes hepatic polyploidy [26–28]. Furthermore, *E2f8*, a gene whose expression was day-night reversed by 4T1 transplantation, is a known regulator for hepatic polyploidy [28] (Figure 4H). These led us to examine hepatic polyploidy in 4T1-bearing mice with the aid of PI staining followed by flow cytometric analyses. In our hands, diploid (2n) and tetraploid (4n) hepatocytes were predominant whereas 8n cells were barely detected both in the sham and 4T1-transplanted group (Figure 5G). We found that the population of tetraploid cells (4n) increased in the liver of 4T1-bearing mice (Figure 5G). In addition, hepatocytes in 4T1-bearing mice appeared to be enlarged (Figure 5B). These data were consistent with the fact that the size of hepatocytes correlated with polyploidy [26, 29]. To our knowledge, enhanced hepatic polyplodization has not been fully appreciated as a phenotype induced by distant cancer to date. In conclusion, we found that 4T1 transplantation generated systemic cancer signals that specifically altered liver physiology, causing inflammation, increasing oxidative stress and polyploidy.

## DISCUSSION

Circadian gene expression programs are critical for organisms to adjust their physiology to daily events. Utilizing comprehensive transcriptome analyses, we present that 4T1 breast cancer cells impair daily expression patterns of hepatic circadian genes. Compared to the recently published results showing that the lung adenocarcinoma rewires hepatic circadian transcriptome [17], our results are unique in that 4T1 cells disrupted expression of core clock transcription factors specifically (Figures 1 and 2). Additionally, we discovered a particular pattern of disruption, the day-night reversal for e.g *E2f8* (Figure 4H). The rewired pattern of circadian gene expression may underlie increased oxidative stress and anomalous polyploidy in the liver of 4T1-bearing mice (Figure 5H).

### Possible mechanisms underlying the hepatic circadian disruption caused by 4T1 transplantation

We sought for a possible logic that could explain the disrupted circadian gene expression (Figures 2-5) on the basis of the altered 7 core clock genes (Figure 2) in the liver of 4T1-bearing mice. Ueda and colleagues previously proposed a simple principle that underlies rhythmic gene expression [6]. A D-box-controlled (i.e. “day-time”) activator(s) (e.g. Rorγ) and E/E'-box controlled (i.e. “morning”) repressor(s) (e.g. Rev-erbα) act together on the RRE-element to form “night-time” transcriptome. In parallel, an E/E'-box-controlled (i.e. “morning”) activator(s) (e.g. Dbp) and RRE-controlled (i.e. “night-time”) repressor(s) (e.g. E4bp4) regulate D-box-mediated gene expression to establish day-time transcriptome.

Mapping the obtained phenotypes in core clock gene expression to the Ueda's modeling [6] gave rise to several hypotheses. The delayed activation and rapid repression of *Rorγ* (Figure 2C) may theoretically cause (a) delayed activation and/or rapid repression of Rorγ-RRE-controlled “night-time” genes. Indeed, this type of abnormality was observed for three of the three “night-time” core clocks surveyed (*Bmal1*, *Cry1*, and *E4bp4*) (Figure 2). Furthermore, night-time genes represented by *Sdr9c7*, *Rdh11*, *Elovl3* and *Osgin1* exhibited clear phase-shifts (Figures 4D, 4E, 4G and Supplementary Figure 3C). These implicated a role for the abnormal rhythm of *Rorγ* in disrupting a set of night-time gene expression. The reduced expression of *Rev-erbα* during day-time may result in (b) insufficient repression of RRE-controlled genes. The disruption pattern of *Rev-erbα* can be considered also as a phase-advance, possibly affecting peak times of night-time genes. Combination of (a) and (b) may affect amplitudes of night-time gene expression (despite not necessarily). Considering that these two genes were ranked within the top3 among the differentially expressed core clock genes in the liver, night-time gene expression was likely to be more severely affected by 4T1 transplantation. This notion was supported by the RNA-seq and qRT-PCR data (Figure 4A): night-time genes looked preferentially affected.

The phase advance of *E4bp4* expression with the rapid dropping at ZT18-ZT22 may lead to (c) phase advancement of day-time genes (repression on day-time genes ends earlier). Consistent with this, expression of a day-time gene *Nampt* was phase–advanced (Figure 4F). In addition to the Ueda's modeling [6], the aberrant expression pattern of *Clock* should be taken into account (Figure 2E). The decreased expression of *Clock*/*Bmal1* at ZT22 probably (d) reduces the extent of E/E'-box activation in the morning. The increased expression of *Clock* at ZT14 may (e) wrongly activate morning genes at night (ZT18 and ZT22). (d) and (e) theoretically could buffer with each other, making no alteration in their downstreams. Yet, severer phenotypes in *Rev-erbα* and *Rorγ* led us to assume that these two transcription factors in the liver are the primary targets of 4T1 breast cancer cells. Together, our data suggest that the Rorγ/Rev-erbα-mediated night-time circuit and Clock/Bmal1/E4bp4-dependent day-time pathway were rewired in a liver-specific manner (Figures 2, 4 and Supplementary Table 21).

Understanding how a specific set of genes (*E2f8*, *Nlrp12*, and *Cyp8b1*) completely lost their original expression patterns is of particular interests (Figures 4H-4J). Most strikingly, *E2f8* and *Nlrp12* showed a distinct pattern of disruption, “day-night reversal” (Figure 4H and 4I). These extreme changes were probably caused by combination of (a)-(e). Indeed, Rorγ and Rev-erbα co-occupied the genomic loci of these genes (Supplementary Figure 4). Involvement of these core clocks on the regulation of genes that lost their original rhythm was in part supported by previously published data. For instance, it is known that *Rorγ* KO severely reduced expression of *Cyp8b1* in the liver [30]. Thus, it is likely that the deregulated *Rorγ* expression (Figure 4C) affected *Cyp8b1* expression. Simply because Rev-erbα binds the *Cyp8b1* locus (Supplementary Figure 4C), insufficient repression by *Rev-erbα* likely up-regulates *Cyp8b1* expression. Combination of these two may explain how *Cyp8b1* lost its rhythm.

In summary, we expect that the complexly rewired expression of core clock transcription factors is a cause of downstream disruption. Moreover, it should be noted that multiple inputs such as inflammatory signals may also affect hepatic circadian transcriptome. However, we yet do not understand why only a particular set of circadian genes was affected in the liver of 4T1-bearing mice.

### Possible physiological outcomes of the circadian disruption in the liver of 4T1-bearing mice

One important issue is whether the hepatic circadian disruption affects the liver physiology. A recent study suggested that knockout for *Bmal1*, a core clock factor, resulted in altered mitochondrial homeostasis and increased oxidative stress. A basis for this is that the liver of *Bmal1* KO exhibited the reduced levels of OXPHOS proteins [31]. The authors using ChIP-seq showed that *Bmal1*, *Cry1*, and *Clock* cooperatively regulate expression of OXPHOS genes. Consistent with these, we found several evidences that the liver of 4T1-bearing mice was under increased oxidative stress (Figures 5C-5F). Importantly, 4T1 cancer cells disrupt expression patterns of *Bmal1*, *Cry1*, and *Clock* in the liver (Figure 2), a possible upstream of the decreased OXPHOS expression.

We also found an intriguing coincidence: the liver of 4T1-bearing mice harbored the increased number of polyploid cells (Figure 5G), which has not been recognized as a cancer-induced phenotype to date. A previous study suggests that non-alcoholic fatty liver promotes hepatic polyploidy, which is thought to be mediated by increased oxidative stress [31]. Thus, increased oxidative stress that was possibly due to the altered *Bmal1* expression may promote hepatic polyploidy in the liver of 4T1-bearing mice (Figure 2D). Alternatively, the day-night reversed expression of *E2f8* may cause this phenotype (Figure 4H). Further examinations are required to determine if the deregulated *E2f8* expression and/or Bmal1-dependent oxidative stress are a direct cause of the altered hepatic polyploidy (Figure 5H). Given that the biological role of hepatic polyploid cells is still not well-defined [26, 27], solving these questions is also important to uncover general significance of enhanced hepatic polyploidy in the presence of cancer.

## CONCLUSION

Our comprehensive analyses identify unique patterns of rewired circadian transcriptome in the liver of 4T1 breast cancer-bearing mice. Furthermore, the transcriptome data guided us to demonstrate that the liver of 4T1-bearing mice suffered from oxidative stress, and notably, increased hepatic polyploidy. Three important issues remain unsolved. Core clock transcription factors typically control thousands of genes [7, 8]. It is currently unclear why only a set of genes was affected by 4T1 transplantation. Moreover, the causative role of the rewired circadian gene expression in the liver pathologies caused by 4T1 needs to be clarified (Figure 5H). For this, it is essential to disrupt the rhythmic expression of specific circadian gene(s) *in vivo*. Identification of a specific factor contributing to the liver pathologies will be also useful for unveiling physiological importance of them (e.g. in hepatic polyploidy). Importantly, why 4T1 cancer cells pay a cost for this phenomenon is unanswered. Are there any benefits for cancer cells to disrupt host clocks in the liver? Answering this question requires a mutant cancer cell line completely defective of interfering with hepatic circadian transcriptome. The current study provides a comprehensive platform for addressing these questions. Our work will be useful to understand how cancer alters host physiology, and ultimately, to target disrupted host physiology for potentially better quality of life for cancer patients.

## MATERIALS AND METHODS

### Cell culture

4T1 cells were cultured in 10 cm dishes in RPMI containing 10% fetal bovine serum and 1% penicillin/streptomycin and incubated in a 37°C, 5% CO_2_ tissue culture incubator.

### Mice

All animal protocols were approved by the Animal Care and Use committee of Advanced Telecommunications Research Institute International (permission numbers: AN20150002 and AN20160002). 7˜9 week old female BALB/c mice were purchased from Japan SLC, Inc. (Japan) and entrained at least for 1 week before experiments. Mice were housed in a 12-hour light/dark paradigm with food and water available *ad libitum*.

### 4T1 cancer transplantation

2nd recipient mice used in Figure 1 were prepared by the following procedures. 4T1 cancer cells were washed with PBS and trypsinized from a culture plate, and resuspended with serum free RPMI medium. Mice were anesthetized with isoflurane, and 4T1 cancer cells were subcutaneously injected into mammary gland using a 1 ml syringe attached with a 30 G needle (5×10^5^ cells in 100 μl serum free RPMI). At 7 days after injection, mice were sacrificed to obtain cancer tissues, which were cut into uniform-sized pieces (3 mm × 3 mm x 3 mm) to be transplanted into mammary gland of 2nd recipient BALB/c mice.

For preparing 1st recipient mice used in Figures 2-5, 4T1 cells were washed with PBS and trypsinized from a culture plate and transferred to a 50 ml tube. After washing with serum free RPMI medium, cells were centrifuged for 5 min at 2,000 rpm, and the supernatant was discarded. The pellets were resuspended with serum free RPMI medium, and final cell concentration was adjusted to 1×10^7^ cells/ml. Mice were anesthetized with isoflurane, and inoculated with 4T1 cancer cells (1×10^6^ cells in 100 μl serum free RPMI). 4T1 cancer cells were subcutaneously injected into mammary gland using a 1 ml syringe attached with a 30 G needle. As a sham-operation, mice were inoculated with the same volume of serum free RPMI.

### RNA isolation, cDNA synthesis, and quantitative reverse transcription PCR (qRT-PCR)

Mice organs were crushed in liquid-nitrogen and homogenized with Trizol reagent (Thermo Fisher Scientific, USA). The obtained supernatants were further purified with RNeasy mini kit (Qiagen, Netherlands) according to manufacture's instruction. 30-100 ng of total RNA was reverse-transcribed with Transcriptor First Strand cDNA synthesis kit (Roche, Switzerland). The obtained cDNA was 10-fold diluted with water and subjected to qRT-PCR (10 μl per reaction), which were performed using the LightCycler 480 Instrument II System and SYBR Green Master Mix (Roche, Switzerland). *B2M* was used as an internal standard. The sequences of primers used in qRT-PCR are shown in Supplementary Table 22.

### RNA-seq and bioinformatic analysis

RNA-seq analyses were performed using tophat2 [32], cufflinks, and cuffdiff [33]. Statistical significance of the observed gene expression differences was judged by cuffdiff [33]. When comparing two scores in two datasets (data1 vs data2 (e.g. sham vs 4T1)), we restricted our analyses to genes showing (i) FPKM in data1 > 0 and FPKM in data2 > 0 and (ii) FPKM in data1 ≥ 1 or FPKM in data2 ≥ 1. These enabled us to exclude lowly expressed genes in comparing datasets of interests. The list of cycling genes was obtained using JTK cycle software [23]. To avoid “less-reliable” circadian genes in the following analyses, we extracted 329 genes that were considered to be rhythmic both in our dataset (Supplementary Table 11) and [7]. We added three genes (*E2f8*, *Osgin1*, and *Nnmt*) to the list even though these were listed only either in Supplementary Table 11 or [7] since our qRT-PCR data well validated these as circadian genes. These analyses defined in total 332 genes as most reliable cycling genes (Supplementary Table 12). RNA-seq data published in the present study have been deposited under the accession number of DRA005205. In-house R scripts used for processing data are all available upon request. Gene ontology analyses were done with http://geneontology.org/page/go-enrichment-analysis. For investigating genomic occupancy of Rev-erbα and Rorγ, wig-formatted data were retrieved from GSM1659694 and GSM840528 to be visualized by the IGV viewer [22, 34].

### Circular statistical analysis

Peak times of circadian gene expression were estimated by fitting a sinusoidal function to the qRT-PCR data [24]. The sinusoidal function used in the analysis is given by y(t) = b + A cos{2π(t − p)/24}, where y(t) is the gene expression level at time point t [h], b and A are the mean and amplitude of gene expression, respectively, and p [h] is the peak time (0 ≤ p < 24). For a given circadian gene, the set of parameters (b, A, p) is estimated by nonlinear least-square regression. Let (bS, AS, pS) and (bT, AT, pT) denote the sets of estimated parameters for Sham and 4T1, respectively. Then, the estimated peak difference between Sham and 4T1 is obtained as Δ = pS − pT (−12 ≤ Δ ≤ 12). The results are summarized in Supplementary Table 23.

### Western blotting

The expression levels of subunit proteins in mitochondrial respiratory complexes were analyzed by western blotting with Total OXPHOS Rodent WB Antibody cocktail (1:250, ab110413, abcam, US). As the secondary antibody, anti-mouse IgG (1:20000, 113-035-174, Jackson ImmumoResearch, USA) was used. Signals were visualized with ECL plus Western Blotting Detection Reagents (GE Healthcare, Japan) and analyzed by CCD digital imaging system LAS-4000 Luminescent Image Analyzer (GE Healthcare, Japan).

### Flow cytometry

Fresh liver cell suspension was fixed in 70% ethanol overnight at 4°C. Cells were washed with PBS and centrifuged for 5 min at 400 g, and treated with Pepsin (0.5 mg/ml in 0.1 N HCl) for 20 min at room temperature to isolate hepatocyte nuclei. After centrifugation (1000 g for 5 min), cells were resuspended with 2 N HCl and incubated for 12 min at 37°C. The nuclei were stained with buffer containing propidium iodide (PI) and RNase (250 μg/ml), and analyzed with EC800 cell analyzed (Sony, Japan).

### Histochemistry

Dissected livers were fixed in 10% formalin neutral buffer solution (Wako) at room temperature until use. Fixed livers were dehydrated by dilution series of ethanol (70, 80, 90, 99.5 and 100%) and xylene. Paraffin filtration was performed at 65°C for overnight and then samples were embedded in the paraffin at room temperature. Paraffin sectioning (thickness = 5 μm) was performed with HM 340E Rotary Microtome (Thermo Fisher SCIENTIFIC). The sections were deparaffinized by xylene and ethanol treatments and then stained with Mayer's Hematoxylin and eosinY (Wako Pure Chemical Industries). Sample images were taken by using Nikon ECLIPSE Ni-E.

## SUPPLEMENTARY MATERIALS FIGURES AND TABLES






