# Supplementary material for: Remote reprogramming of hepatic circadian transcriptome by breast cancer
Source: Oncotarget. 2017 Apr 6;8(21):34128–40. doi: 10.18632/oncotarget.16699 (PMC5470956; doi:10.18632/oncotarget.16699)
Supplement: Supplementary file 1 [file oncotarget-08-34128-s001.pdf]

# Remote reprogramming of hepatic circadian transcriptome by breast cancer

## SUPPLEMENTARY MATERIALS

## SUPPLEMENTARY FIGURES AND TABLES

## REFERENCES

1. Cho H, Zhao X, Hatori M, Yu RT, Barish GD, Lam MT, Chong LW, DiTacchio L, Atkins AR, Glass CK, Liddle C, Auwerx J, Downes M, et al. Regulation of circadian behaviour and metabolism by REV-ERB-alpha and REV-ERB-beta. Nature. 2012; 485: 123-127.

2. Zhang Y, Fang B, Emmett MJ, Damle M, Sun Z, Feng D, Armour SM, Remsberg JR, Jager J, Soccio RE, Steger DJ, Lazar MA. GENE REGULATION. Discrete functions of nuclear receptor Rev-erbalpha couple metabolism to the clock. Science. 2015; 348: 1488-1492.

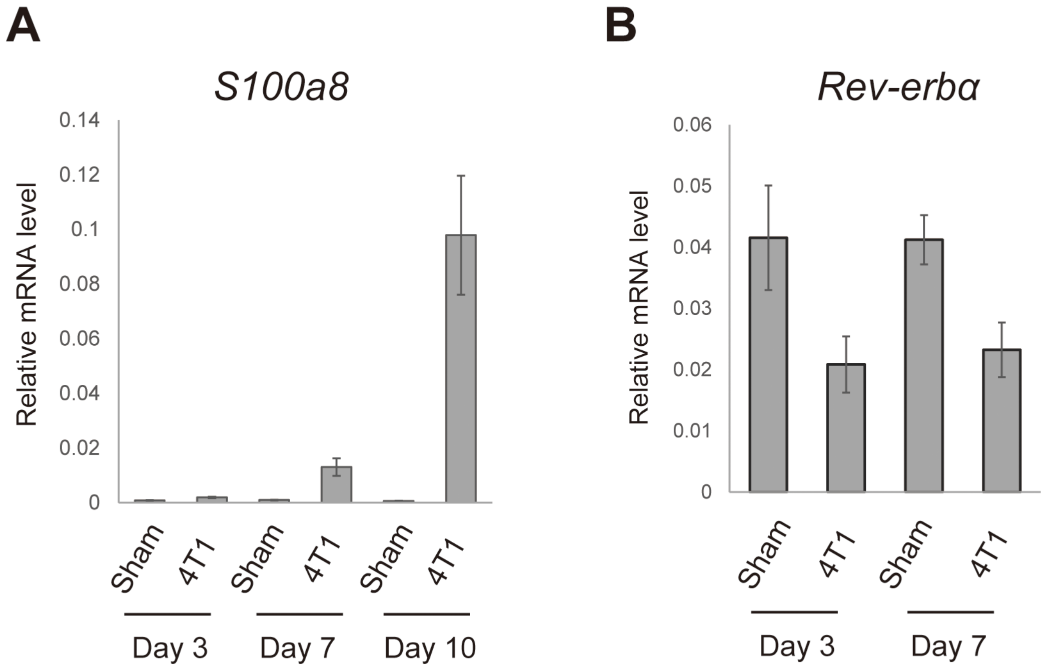


**Supplementary Figure 1: Expression of *S100a8* and *Rev-erbα* in the liver of tumor-bearing mice.** **(A)** Expression of *S100a8* gene in the liver of sham-operated (Sham) and 4T1-bearing (4T1) mice on the indicated time points, determined by qRT-PCR. **(B)** Expression of *Rev-erbα* gene in the liver of sham-operated (Sham) and 4T1-bearing (4T1) mice, determined by qRT-PCR on the indicated day points. Data are presented as the mean ± SE (n = 8).

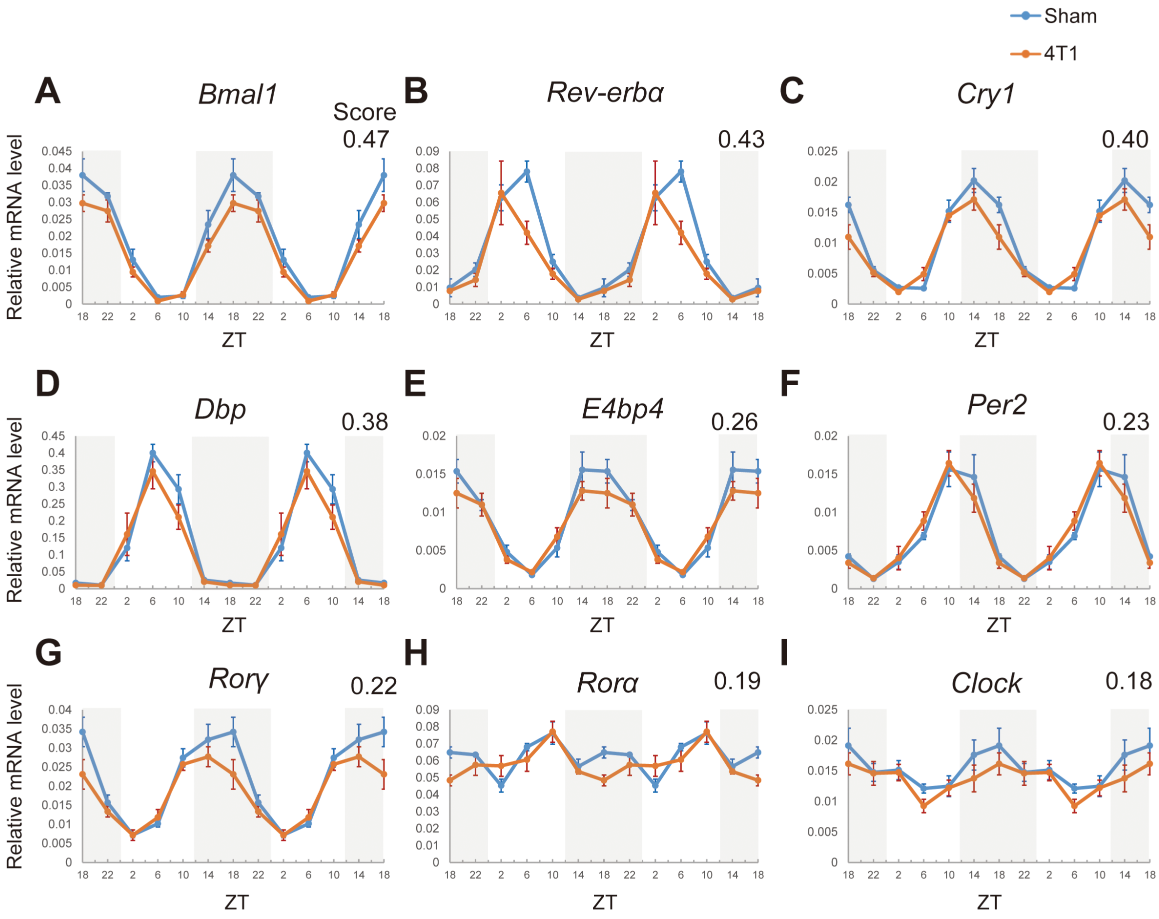


**Supplementary Figure 2: Daily gene expression patterns of the core clock genes in the kidney of 4T1-bearing mice.** **(A)-(I)** Daily expressions of the core clock genes, *Rev-erbα*, *Clock*, *Bmal1*, *Per2*, *Cry1*, *Rorα*, *Rorγ, Dbp*, and *E4bp4* in the kidneys of sham-operated (Sham) and 4T1-bearing (4T1) mice on 7 dpt, as determined by qRT-PCR. Daily expression patterns are double-plotted. Data are presented as the mean ± SE (n = 6). Average value of absolute $\log_2$ fold changes is indicated in the upper-right corner.

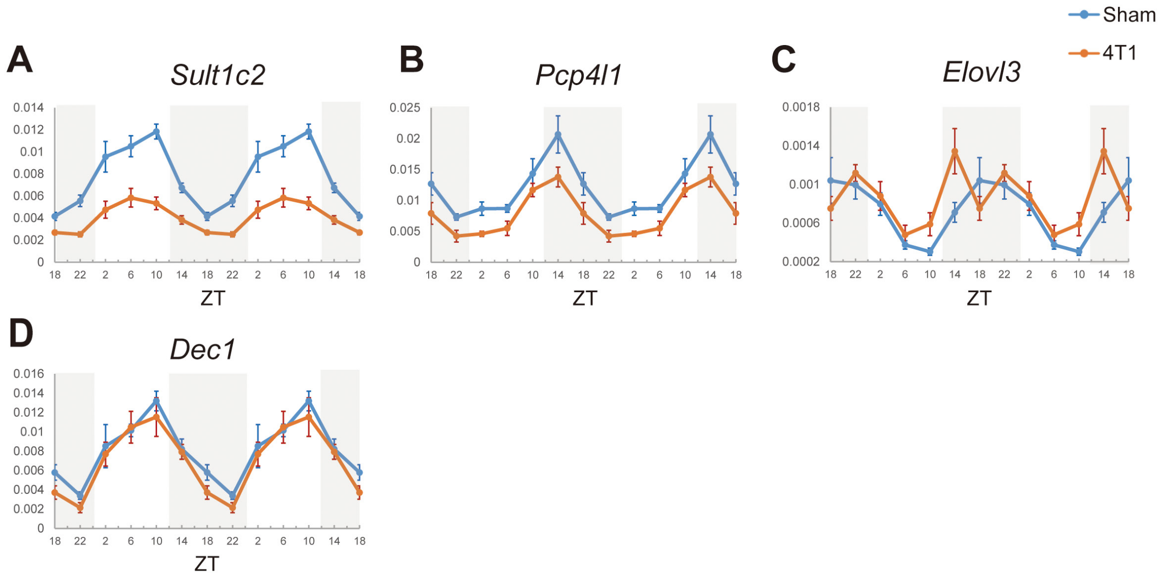


**Supplementary Figure 3: The expression patterns of representative 4T1-affected circadian genes in the liver of 4T1-bearing mice. (A)-(C)** Daily expression of *Sult1c2*, *Pcp4l1*, *Elovl3*, and *Dec1* in the liver of sham-operated (Sham) and 4T1-bearing (4T1) mice, as determined by qRT-PCR. Mice were sacrificed on 7 dpt at indicated time points. Daily expression patterns are double-plotted. Data are presented as the mean ± SE (n = 8 for ZT22 of the sham sample of *Dec1*, n = 9 for ZT18, 2, 6, 10, 14 of the sham samples of *Dec1*, and n = 6 for other samples).

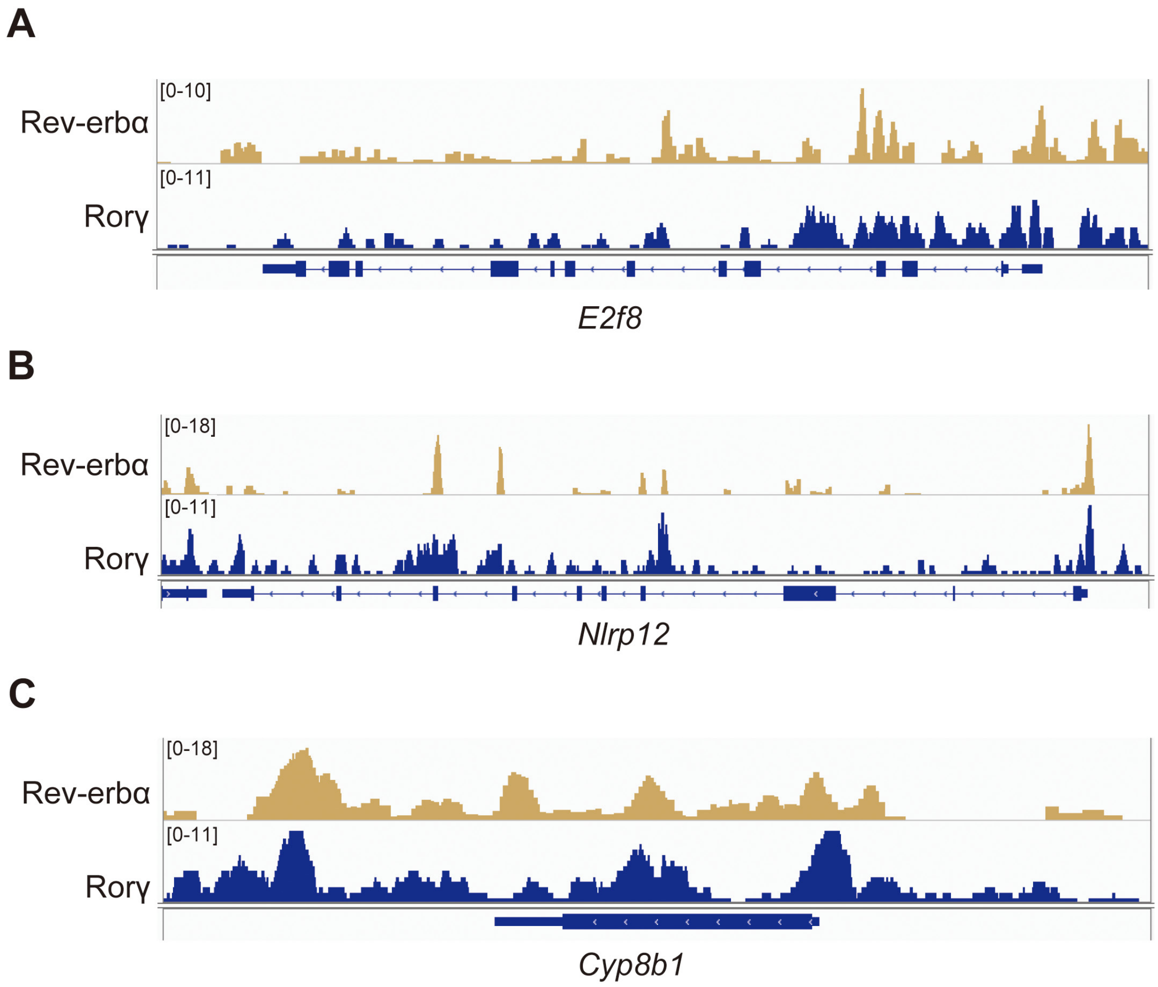


**Supplementary Figure 4: The occupancy of Rev-erbα and Rorγ in the genomic locus of *E2f8*, *Nlrp12*, and *Cyp8b1*.** **(A)-(C)** Data from GSM1659694 and GSM840528 are visualized using the IGV viewer (Cho et al., 2012; Zhang et al., 2015).

**Supplementary Table 1: The result of cuffdiff comparing the liver transcriptome of Sham-operated and 4T1-bearing mice (data from D3 and D7 post transplantation)**

See Supplementary File 1

**Supplementary Table 2: The list of liver genes significantly affected by 4T1-transplantation**

See Supplementary File 1

**Supplementary Table 3: The result of cuffdiff comparing the lung transcriptome of Sham-operated and 4T1-bearing mice (data from D3 and D7 post transplantation)**

See Supplementary File 1

**Supplementary Table 4: The list of lung genes significantly affected by 4T1-transplantation**

See Supplementary File 1

**Supplementary Table 5: The result of cuffdiff comparing the heart transcriptome of Sham-operated and 4T1-bearing mice (data from D3 and D7 post transplantation)**

See Supplementary File 1

**Supplementary Table 6: The list of heart genes significantly affected by 4T1-transplantation**

See Supplementary File 1

**Supplementary Table 7: The result of cuffdiff comparing the kidney/adrenal gland transcriptome of Sham-operated and 4T1-bearing mice (data from D3 and D7 post transplantation)**

See Supplementary File 1

**Supplementary Table 8: The list of kidney/adrenal gland genes significantly affected by 4T1-transplantation**

See Supplementary File 1

**Supplementary Table 9: The list of RPKM scores of the genes expressed in the liver of sham-operated mice (ZT02/ZT06/ZT10/ZT14/ZT18/ZT22)**

See Supplementary File 1

**Supplementary Table 10: The list of RPKM scores of the genes expressed in the liver of 4T1-bearing mice (ZT02/ZT06/ZT10/ZT14/ZT18/ZT22)**

See Supplementary File 1

**Supplementary Table 11: The result of JTK cycle on 12 samples from sham-operated mice (genes showing $p < 0.05$ and AMP > 0 are shown)**

See Supplementary File 1

**Supplementary Table 12: The list of 332 genes considered to be circadian genes based on two independent datasets (ours and Vollmers et al. 2012)**

See Supplementary File 1

**Supplementary Table 13: The list of log2 fold change scores (4T1/Sham) at each ZT for 182 non-circadian genes that are significantly affected by 4T1 at all ZTs**

See Supplementary File 1

**Supplementary Table 14: The list of the liver genes significantly affected by 4T1-transplantation at ZT02**

See Supplementary File 1

**Supplementary Table 15: The list of the liver genes significantly affected by 4T1-transplantation at ZT06**

See Supplementary File 1

**Supplementary Table 16: The list of the liver genes significantly affected by 4T1-transplantation at ZT10**

See Supplementary File 1

**Supplementary Table 17: The list of the liver genes significantly affected by 4T1-transplantation at ZT14**

See Supplementary File 1

**Supplementary Table 18: The list of the liver genes significantly affected by 4T1-transplantation at ZT18**

See Supplementary File 1

**Supplementary Table 19: The list of the liver genes significantly affected by 4T1-transplantation at ZT22**

See Supplementary File 1

**Supplementary Table 20: The list of 96 out of 332 circadian genes showing a significant change at least at one ZT**

See Supplementary File 1

**Supplementary Table 21: Possible hypotheses that explain the disrupted circadian gene expression**

See Supplementary File 2

**Supplementary Table 22: List of qPCR primers used in this study**

See Supplementary File 1

**Supplementary Table 23: The acrophases of all circadian genes tested by qRT-PCR in this study**

See Supplementary File 1
